# Thin anterior uterine wall with incomplete uterine rupture in a primigravida detected by palpation and ultrasound: a case report

**DOI:** 10.1186/1752-1947-5-14

**Published:** 2011-01-17

**Authors:** Shigeki Matsubara, Kazuhiko Shimada, Tomoyuki Kuwata, Rie Usui, Mitsuaki Suzuki

**Affiliations:** 1 Department of Obstetrics and Gynecology and Jichi Perinatal Education Center, Jichi Medical University, 3311-1 Yakushiji, Shimotsuke, Tochigi 329-0498, Japan

## Abstract

**Introduction:**

Uterine rupture is an obstetric complication associated with significant maternal and fetal morbidity and mortality. This disorder usually occurs with a scarred uterus, especially in a uterus with prior Cesarean section. Uterine sacculation or diverticulum may also lead to a thin uterine wall during pregnancy.

**Case presentation:**

A 27-year-old Japanese primigravid woman was admitted to our hospital due to weak, irregular uterine contractions in her 38^th ^week of gestation. She had no past history of uterine surgery or known diseases. A hard mass was palpable in her abdomen. An ultrasound revealed that the anterior uterine wall was thin and bulging, with a fetal minor part beneath it which corresponded to the palpated mass. A Cesarean section was performed which revealed a thin anterior uterine wall with incomplete uterine rupture. The woman and baby were healthy.

**Conclusions:**

Although extremely rare, an unscarred primigravid uterus can undergo incomplete rupture even without discernable risk factors or labor pains. Abdominal palpation and ultrasound may be useful in detecting this condition.

## Introduction

Uterine rupture is an obstetric complication associated with significant maternal and fetal morbidity and mortality. This disorder usually occurs in a scarred uterus, especially secondary to prior Cesarean section, and is therefore considered a disease of multigravida [1,2]. A few reports have indicated that a uterine rupture can occur in primigravida, although this is extremely rare [2,3], with etiological or risk factors including a history of uterine surgery, labor augmentation or underlying connective tissue disease [2-4]. A thin uterine wall, as a result of uterine sacculation [5,6] or uterine diverticulum [7], may also induce uterine rupture.

We report the case of a primigravid woman with a thin anterior uterine wall; a feature compatible with incomplete uterine rupture. Underlying etiological factors were indiscernible. Her condition was detected by abdominal palpation and then ultrasound. This case report suggests that an unscarred primigravid uterus can show the features of incomplete rupture even in the absence of discernable risk factors and that abdominal palpation and ultrasound may be useful in diagnosis.

## Case presentation

**Figure 1:**
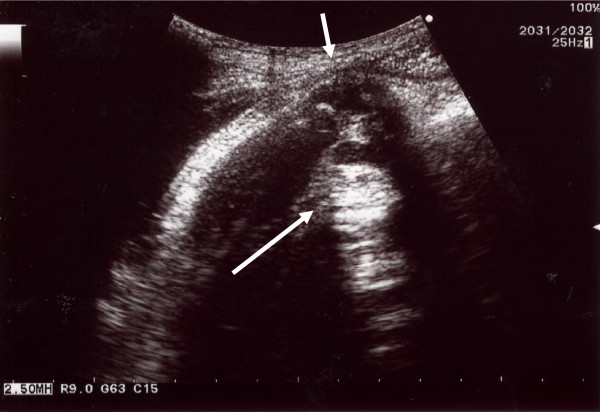
**An abdominal ultrasound image of the uterine wall and the fetal minor part**. The small arrow indicates a thin uterine wall, which is slightly bulging. Beneath the thin uterine wall a fetal minor part (large arrow) is visible, which was palpated as a hard mass through the abdomen.

A 27-year-old Japanese primigravid woman was admitted to our hospital because of slight uterine contractions at 38^+6 ^weeks of gestation. Her past history was unremarkable. She had received no uterine surgery or procedure. There were no symptoms or history suggesting the presence of Ehlers-Danlos syndrome in either herself or her family members. We did not perform drug screening, including cocaine (a possible cause of uterine rupture). She had received regular pregnancy checks on up to 14 occasions and an ultrasound examination had been performed at every visit, according to our institute protocol. An ultrasound had revealed no abnormal uterine structure, although special attention had not been paid to the uterine wall. On admission, a speculum examination revealed that her cervix was normally positioned and not ventrally located. A digital examination revealed a cervical ostium opening of 2.0 cm with 1.5 cm effacement. A cardiotocogram showed a normal fetal heart rate (FHR) pattern with weak uterine contractions of 20 second duration once per hour. There was no tenderness or palpable mass. An attending doctor performed an ultrasound examination, which revealed normal placentation without myoma and an amniotic fluid index of 15 cm (normal range: 5-25 cm). However, he did not comment on the uterine wall thickness. Although she preferred to remain in hospital, the irregular contractions occurring only once per hour led to her decision to discharge at 39^+2 ^weeks of gestation. During her time in our hospital, a cardiotocogram was performed six times and mild variable decelerations with normal variability were observed on two occasions. Otherwise, FHR patterns were normal. Prior to her discharge, the cervical ostium opening was 3.0 cm with 1.0 cm effacement, with -2 station of the presenting part, the head. This roughly indicated that active labor had not yet begun. The attending doctor palpated her abdomen and incidentally noted a hard thumbhead-sized mass protruding at the midline, 10 cm below her umbilicus. An abdominal ultrasound revealed a bulging thin uterine wall of <1 mm with a fetal minor part beneath it (Figure 1), which corresponded to the hard palpable mass. Slight tenderness was observed at the thin uterine wall. Although primigravidity, an unscarred uterus and the absence of regular contractions reduced the possibility of an impending uterine rupture, ultrasound findings led us to suspect it and an emergent Cesarean section was performed.

**Figure 2:**
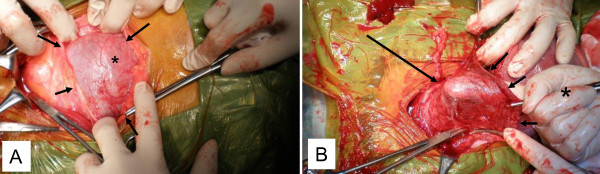
**Operative findings during a Cesarean section**. A: The asterisk indicates the thin anterior uterine wall. Arrows indicate the peritoneum. The left side of the photograph is the caudal side of the patient. B: After delivering the fetus and removing the placenta, the thin wall was gently pushed from inside the uterus with a finger. The finger tip (large arrow) is clearly visible through the thin wall. The asterisk indicates the surgeon's right hand. Small arrows indicate the uterine incision site.

Her vesicouterine fold was located in the normal position and her bladder did not overlay the lower uterine segment. Cephalad to the vesicouterine fold, an approximately 8 × 8 cm area of the uterine anterior wall was thin and a fetal hand and head were visible through it (Figure 2A and 2B). A transverse incision was made cephalad to this thin wall area, and a 2604 g (appropriate-for-date according to the Japanese standard) female baby was delivered with Apgar scores of 8 and 9 at one to five minutes, respectively. The placenta was delivered spontaneously. We excised the thin part of the uterine wall and reconstructed the site with a total blood loss of 370 ml. The uterine body was located normally, without distortion or posterior incarceration. A histological examination of the excised uterine wall was not performed. An abdominal and vaginal ultrasound seven days post-partum revealed a well-involuted uterus and the absence of discernable uterine anomalies. Her post-partum course was unremarkable. Considering that a short inter-delivery period of <18 months would increase the risk of rupture of the scarred uterus [8], we advised her to avoid further pregnancy for at least one year. The patient and her baby were healthy six months after the birth.

## Discussion

This case suggested two clinical issues. Firstly, an unscarred pre-labor primigravid uterus can show a very thin uterine wall, compatible with incomplete uterine rupture, without apparent etiological or risk factors. Secondly, abdominal palpation and ultrasound may be useful to detect this condition even in the absence of symptoms or signs.

Uterine ruptures are divided into complete and incomplete. In the former, the uterine serosa together with the uterine muscular layer is perforated and thus the amniotic cavity directly communicates with the abdominal cavity. In the latter, the uterine muscular layer is lost but the uterine serosa is preserved. Incomplete rupture is also referred to as uterine dehiscence. Data obtained from cases of complete rupture of a primigravid unscarred uterus may be applicable to, or may at least provide information on, incomplete rupture. Therefore, our discussion is based on data on complete rupture of a primigravid unscarred uterus, since reports focusing on incomplete rupture of a primigravid unscarred uterus are lacking. Two disorders, uterine sacculation and uterine diverticulum, may also induce a thin uterine wall, thus causing uterine rupture. While uterine rupture, complete or incomplete, denotes the final uterine feature, uterine sacculation and diverticulum denote the disease entities. To avoid confusion, 'rupture' has been used to denote the final uterine condition, if not specified. Sacculation and diverticulum will be touched on separately.

The first important clinical issue is that an unscarred pre-labor primigravid uterus can show a very thin uterine wall, compatible with incomplete uterine rupture, without apparent etiological or risk factors. Walsh and Baxi [2], reviewing the literature over six decades (1946-2006), found 36 primigravid uterine ruptures, and we have found a further 21 cases [3,9-12]. Of these 21 cases, 15 were reported in a case series from Nepal [11], with all ruptures occurring after labor of >48 hours, and 12 having received no antenatal care. Of 57 (36+21) cases previously reported, 55 women had some discernable etiological or risk factors for rupture, including a past history of uterine surgery, congenital uterine anomaly, adherent placenta, labor, or oxytocin and/or prostaglandin use [2-4,9-12]. The etiological factors were described as indiscernible in the remaining two cases; one of which was described in a case series and lacked detailed data [1]. In the remaining case - that of a 21-year-old Indian woman - she had stated that she was primigravida, but she had received no antenatal care until rupture and no further evaluation to identify any underlying condition was performed [13]. Therefore, the data was insufficient to claim 'unknown etiology'. There have been no reported cases of primigravid unscarred uterine rupture of unknown etiology, employing its strict definition. Etiological or risk factors were not discernable in our case report. She had no past uterine surgery, no detectable uterine anomalies and no abnormal placentation. Her family and past history rejected the possibility of Ehlers-Danlos syndrome, which is reported to cause primigravid uterine rupture [4]. She experienced weak uterine contractions, false pains for approximately 86 hours; however, they were very weak and may not have been the cause of the rupture.

Another possible cause of a thin uterine wall is uterine muscular weakness. Uterine sacculation or uterine diverticulum may lead to uterine wall weakness. Uterine sacculation is defined as a transitory pouch or sac-like structure developing from a portion of the gravid uterus [6]. The typical form of sacculation results from an incarcerated retroverted uterus [5,6]. A ventrally-located cervical ostium and vagina may cause physicians to suspect this diagnosis. Magnetic resonance imaging (MRI) may provide a pre-operative diagnosis [5]. In this condition, the anterior uterine wall becomes stretched and thinned. The bladder and uterine cervix are also stretched and a Cesarean section without diagnosis may present difficulties in identifying the bladder and cervix, and therefore in opening the lower uterine segment [5]. In our case report, the diagnosis of this type of uterine sacculation, accompanied by incarcerated retroverted uterus, can be precluded. Uterine incarceration was absent and her lower uterine segment was located in the normal position. Other conditions, such as previous surgery, a primary myometrial defect, uterine malformation or placental abnormalities are listed as possible causes of uterine sacculation [6,14]. However, in our case report, these underlying conditions were not identified. We therefore could not determine whether uterine wall weakness of unknown etiology was present in our case report.

Uterine diverticulum is frequently misunderstood and reported as uterine sacculation [7]. Uterine diverticulum has a narrow connection with the uterine cavity and a thicker wall than in sacculation [7]. While uterine sacculation is usually observed during pregnancy [6,14], diverticulum is usually detected in non-pregnant women. Uterine diverticula as complications during pregnancy are rare. Rajiah *et al. *[7] reported a primigravid woman in whom an MRI revealed uterine diverticulum in the 22^nd ^week of gestation. A Cesarean section was performed in the 31^st ^week. The diverticulum originated from the posterolateral wall of the uterine body and did not contain the fetus. The diverticulum was not excised due to surgical risks. In the post-partum period, the diverticulum was observed via ultrasound. The authors considered that the underlying etiology for the diverticulum may have been congenital because this patient was primigravida with no prior cervical or uterine procedure. Sun *et al. *[15] reported a case in which the gestational sac implanted in a diverticulum: the pregnancy was terminated at an estimated seven to eight weeks. The authors considered that abnormal development of the paramesonephric duct may cause a congenital uterine deformity, leading to a formation of diverticulum. In our case report, the thin uterine wall did not show the morphological features of typical diverticulum: the narrow neck characteristic of typical diverticulum was not observed. Thus, this diagnosis may be precluded. Although the morphological features of the thin uterine wall in our case report differed from those of uterine diverticulum, the possible underlying etiology of diverticulum suggested by Sun *et al. *[15] may also hold true here. However, we do not have any evidence. If we had not excised the thin uterine wall, and if this portion had later bulged and eventually made a diverticulum, then the thin uterine wall observed may have proved to be a feature of the future occurrence of uterine diverticulum. The clinical course of uterine diverticulum, especially that of unknown etiology, is not yet clear and further cases are required.

The second important clinical issue is that abdominal palpation and ultrasound may be useful in detecting this condition. Although abdominal pain [2,3] and non-reassuring FHR patterns, especially fetal bradycardia, have been reported to be signs of impending or actual uterine rupture in some cases, our case report showed no abdominal pain and no abnormal FHR pattern, except for mild variable deceleration. It was fortunate that an attending doctor happened to palpate the abdomen even in the absence of symptoms or signs and noticed a hard thumbhead-sized mass. To the best of our knowledge, this is the first reported case in which palpation incidentally revealed a protruding fetal minor part, with ultrasound identifying this condition as impending complete uterine rupture, leading to an emergent Cesarean section.

Congenital weakness of the uterine wall may result in a thin uterine wall and this has been discussed in relation to uterine sacculation and diverticulum. However, it remains undetermined as to why and when the uterine wall becomes thin. Putting aside the etiology of the thin uterine wall observed here, physicians must be aware that an unscarred primigravid uterus can undergo incomplete rupture.

## Conclusions

An unscarred primigravid uterus can undergo incomplete rupture even without etiological or risk factors, or the presence of any symptoms or signs. Abdominal palpation and ultrasound may be useful for diagnosis.

## Abbreviations

FHR: fetal heart rate; MRI: Magnetic resonance imaging

## Consent

Written informed consent was obtained from the patient for publication of this case report and any accompanying images. A copy of the written consent is available for review by the Editor-in-Chief of this journal.

## Competing interests

The authors declare that they have no competing interests.

## Authors' contributions

SM, KS and RU diagnosed, investigated, followed up on and managed the patient's care. SM and TK wrote the manuscript. MS provided important suggestions regarding medical content. All authors read and approved the final manuscript.
